# Complete Genome Sequence of the Cyanogenic Phosphate-Solubilizing *Pseudomonas* sp. Strain CCOS 191, a Close Relative of *Pseudomonas mosselii*

**DOI:** 10.1128/genomeA.00616-15

**Published:** 2015-06-11

**Authors:** Theo H. M. Smits, Joël F. Pothier, Michela Ruinelli, Jochen Blom, David Frasson, Chantal Koechli, Carlotta Fabbri, Helmut Brandl, Brion Duffy, Martin Sievers

**Affiliations:** a Environmental Genomics and Systems Biology Research Group, Institute of Natural Resource Sciences, Zürich University of Applied Sciences, Wädenswil, Switzerland; b Bioinformatics and Systems Biology, Justus-Liebig-Universität, Giessen, Germany; c Microbiology and Molecular Biology Research Group, Institute of Biotechnology, Zürich University of Applied Sciences, Wädenswil, Switzerland; d Institute of Evolutionary Biology and Environmental Studies, University of Zürich, Zürich, Switzerland

## Abstract

We sequenced the complete genome of the isolate *Pseudomonas* sp. CCOS 191. This strain is able to dissolve phosphate minerals and form cyanide. The genome sequence is used to establish the phylogenetic relationship of this species.

## GENOME ANNOUNCEMENT

We recently isolated *Pseudomonas* sp. strain CCOS 191 from a water sample in Zürich, Switzerland, which is able to form large amounts of cyanide and dissolve phosphate minerals. Cyanide-forming microbes, many of which are members of the *Pseudomonas* family (1), are highly present in the environment, at times making up 50% of the soil community (2). Whereas most pseudomonads form cyanide, hypothesized to be a defense mechanism and/or a result of secondary metabolism (3), only in the presence of glycine as direct precursor, *Pseudomonas* sp. CCOS 191 is able to generate cyanide from glucose. Phosphorus is an essential macronutrient for the growth and reproduction of plants and microorganisms and is assimilated mostly from soluble phosphate. In cases in which phosphate-containing rocks (e.g., apatite) are the source of phosphorus in terrestrial environments, minerals have to be converted into a biologically available form (4). *Pseudomonas* sp. CCOS 191 is efficient in the solubilization of phosphate minerals.

Total genomic DNA was isolated according to the method of Pitcher et al. (5). The genome was sequenced using PacBio single-molecule real-time (SMRT) reads (FGCZ, Zurich, Switzerland), as well as >80,000,000 50-bp Illumina paired-end reads (GATC, Konstanz, Germany). Five SMRT cells yielded 371,914 reads, with an average length of 5,416 bp (for a total of 2,014,434,330 bp). These reads were assembled into one contig of 6,030,031 bp using the HGAP approach (6), which, after manual inspection, was found to be circular. To check the assembly and correct for eventual incorrectly assigned bases, a subset of 8,000,000 Illumina reads were mapped against the HGAP unique contig using the Lasergene genomics package (DNAStar, Madison, WI). The final assembly gave a 6,012,947-bp circular chromosome. The sequence was annotated automatically using GenDB (7) with manual optimization, which yielded a total of 5,302 genes.

Based on the phylogeny of the 16S rRNA, *gyrB*, *rpoB*, and *rpoD* housekeeping genes (8), we generated a phylogeny with known members of the *Pseudomonas putida* group, revealing that this strain might represent a novel species. Comparative genomics using EDGAR (9) indicated that the mean average amino acid identity (95.92%) is close to that of *Pseudomonas mosselii* DSM 17497^T^, at the border of the species delineation (10). Based on multilocus sequence analysis (MLSA) data, the closest relative might be *Pseudomonas soli* (11), but no genome sequence of this species is available.

The production of cyanide from glucose may be a phenotypic feature that could be identified using the genome sequence of *Pseudomonas* sp. strain CCOS 191 and exploited for biotechnological applications in the future.

### Nucleotide sequence accession number. 

The nucleotide sequence of the genome of *Pseudomonas* sp. CCOS 191 was deposited at EMBL-EBI under accession no. LN847264.

## References

[1] Knowles CJ, Bunch AW 1986 Microbial cyanide metabolism. Adv Microb Physiol 27:73–111. doi:10.1016/S0065-2911(08)60304-5. 3532718

[2] Faramarzi MA, Stagars M, Pensini E, Krebs W, Brandl H 2004 Metal solubilization from metal-containing solid materials by cyanogenic *Chromobacterium violaceum*. J Biotechnol 113:321–326. doi:10.1016/j.jbiotec.2004.03.031. 15380664

[3] Blumer C, Haas D 2000 Mechanism, regulation, and ecological role of bacterial cyanide biosynthesis. Arch Microbiol 173:170–177. doi:10.1007/s002039900127. 10763748

[4] Browne P, Rice O, Miller SH, Burke J, Dowling DN, Morrissey JP, O’Gara F 2009 Superior inorganic phosphate solubilization is linked to phylogeny within the *Pseudomonas fluorescens* complex. Appl Soil Ecol 43:131–138. doi:10.1016/j.apsoil.2009.06.010.

[5] Pitcher DG, Saunders NA, Owen RJ 1989 Rapid extraction of bacterial genomic DNA with guanidium thiocyanate. Lett Appl Microbiol 8:151–156. doi:10.1111/j.1472-765X.1989.tb00262.x.

[6] Chin C-S, Alexander DH, Marks P, Klammer AA, Drake J, Heiner C, Clum A, Copeland A, Huddleston J, Eichler EE, Turner SW, Korlach J 2013 Nonhybrid, finished microbial genome assemblies from long-read SMRT sequencing data. Nat Methods 10:563–569. doi:10.1038/nmeth.2474. 23644548

[7] Meyer F, Goesmann A, McHardy AC, Bartels D, Bekel T, Clausen J, Kalinowski J, Linke B, Rupp O, Giegerich R, Pühler A 2003 GenDB—an open source genome annotation system for prokaryote genomes. Nucleic Acids Res 31:2187–2195. doi:10.1093/nar/gkg312. 12682369 PMC153740

[8] Mulet M, Gomila M, Scotta C, Sánchez D, Lalucat J, García-Valdés E 2012 Concordance between whole-cell matrix-assisted laser-desorption/ionization time-of-flight mass spectrometry and multilocus sequence analysis approaches in species discrimination within the genus *Pseudomonas*. Syst Appl Microbiol 35:455–464. doi:10.1016/j.syapm.2012.08.007. 23140936

[9] Blom J, Albaum SP, Doppmeier D, Pühler A, Vorhölter F-J, Zakrzewski M, Goesmann A 2009 EDGAR: a software framework for the comparative analysis of prokaryotic genomes. BMC Bioinformatics 10:154. doi:10.1186/1471-2105-10-154. 19457249 PMC2696450

[10] Konstantinidis KT, Tiedje JM 2005 Towards a genome-based taxonomy for prokaryotes. J Bacteriol 187:6258–6264. doi:10.1128/JB.187.18.6258-6264.2005. 16159757 PMC1236649

[11] Pascual J, García-López M, Carmona C, Sousa Tda S, de Pedro N, Cautain B, Martín J, Vicente F, Reyes F, Bills GF, Genilloud O 2014 *Pseudomonas soli* sp. nov., a novel producer of xantholysin congeners. Syst Appl Microbiol 37:412–416. doi:10.1016/j.syapm.2014.07.003. 25097020

